# The association between different insulin resistance indexes and bone health in the elderly

**DOI:** 10.1371/journal.pone.0318356

**Published:** 2025-02-11

**Authors:** Tianjie Lai, Zhihao Su, Rui Chen, Guangan Luo, Sibo Xu, Hangqi Fang, Huanxin Yan, Peng Shen, Konghe Hu

**Affiliations:** 1 Department of Spine Surgery, The Affiliated Yuebei People’s Hospital of Shantou University Medical College, Shaoguan, Guangdong, P.R. China; 2 First Clinical Medical College, Guangdong Medical University, Zhanjiang, China; Bushehr University of Medical Sciences, IRAN, ISLAMIC REPUBLIC OF

## Abstract

The triglyceride-glucose (TyG) index and its related indexes (TyG-BMI, TyG-WC, TyG-WHtR) are effective markers for screening metabolic diseases like insulin resistance (IR). However, few studies have explored the relationship between the TyG and its related indexes with bone density (BMD), osteopenia, and osteoporosis. This is a cross-sectional study that involved 1,303 adults aged 50 years and above from the National Health and Nutrition Examination Survey 2007–2010, and 2013–2014. In the multivariable-adjusted model, linear regression analysis and logistic regression analysis demonstrated that TyG and its related indexes have a significant positive correlation with BMD and a negative correlation with osteopenia/osteoporosis in the femoral neck, lumbar spine, and total hip region. Trend analysis further confirms these associations (p < 0.05). Restricted cubic spline analysis showed a nonlinear relationship between these indexes with BMD and osteopenia/osteoporosis. Sensitivity analyses further confirmed the robustness of these associations. This study reveals the significant and complex correlation between the TyG and its related indexes with BMD and osteoporosis, indicating the potential link between IR and bone health. The TyG and related indexes offer a new perspective for the diagnosis, prevention, and treatment of osteoporosis.

## 1 Introduction

Osteopenia and osteoporosis are metabolic bone diseases characterized by a decrease in BMD and deterioration of bone microarchitecture, leading to increased bone fragility and a significantly higher risk of fractures [1]. Osteopenia and osteoporosis have gradually evolved into an increasingly severe public health issue. It is estimated that currently, about 12.3 million people in the United States have osteoporosis [2], with over 2 million new fracture cases annually, surpassing the total new cases of myocardial infarction, breast cancer, and prostate cancer combined. By 2040, the annual incidence rate is expected to increase by 68%, reaching 3.2 million cases [3]. In the UK, 81% of women aged 50 to 70 suffer from osteoporosis [4]. According to the Osteoporosis Diagnostic Guidelines, the prevalence of osteoporosis increases approximately exponentially in the population after the age of 50 years [4]. As the global population ages, the incidence of osteoporosis and its associated complications is rising, which not only leads to a significant increase in healthcare costs but also emphasizes the urgency of combating osteoporosis. Nonetheless, Osteoporosis, as an asymptomatic and latent disease, often does not exhibit obvious clinical symptoms or biological markers until fractures occur [5]. Consequently, early diagnosis is vital for the effective treatment and identification of individuals at high risk of fractures. The current research focus is on finding new biomarkers to assess the risk factors of osteoporosis. These efforts aim to improve the skeletal health of the global population through efficient assessment of osteoporosis risks, early detection, and effective preventive measures.

Insulin resistance (IR) is due to the disruption of multiple molecular pathways, leading to decreased insulin sensitivity [6,7]. Currently, the gold-standard methods for identifying IR include the hyperinsulinemic-euglycemic clamp technique (HECT) and the Homeostatic Model Assessment of Insulin Resistance (HOMA-IR) [1]. However, due to the complexity and cost of both methods, they are not suitable for routine clinical practice. As a simple and cost-effective alternative, the Triglyceride-Glucose (TyG) index has been shown to have comparable accuracy to HECT and HOMA-IR in assessing IR, and thus it is frequently used in clinical and epidemiologic studies [8–11]. Metabolic syndrome (MetS) is primarily characterized by abdominal obesity and IR [12,13]. Previous studies have shown that insulin resistance, obesity, and MetS are closely related to BMD and osteoporosis [14–17]. Moreover, numerous studies have found that the combination of the TYG index with obesity indices significantly correlates with the risk of various diseases, including psoriasis and cardiovascular diseases [7,18]. However, the relationship between the TyG and its obesity-related indices, such as TyG-Waist Circumference (TyG-WC), TyG-Waist-to-Height Ratio (TyG-WHtR), and TyG-Body Mass Index (TyG-BMI) with osteoporosis has not been adequately studied. Therefore, we conducted a study on the relationship between the TyG and its related indexes with BMD and osteoporosis, providing a new perspective for understanding the underlying mechanisms driving this condition and for the prevention and treatment of osteoporosis.

## 2 Materials and methods

### 2.1 Data source and study population

**Fig 1:**
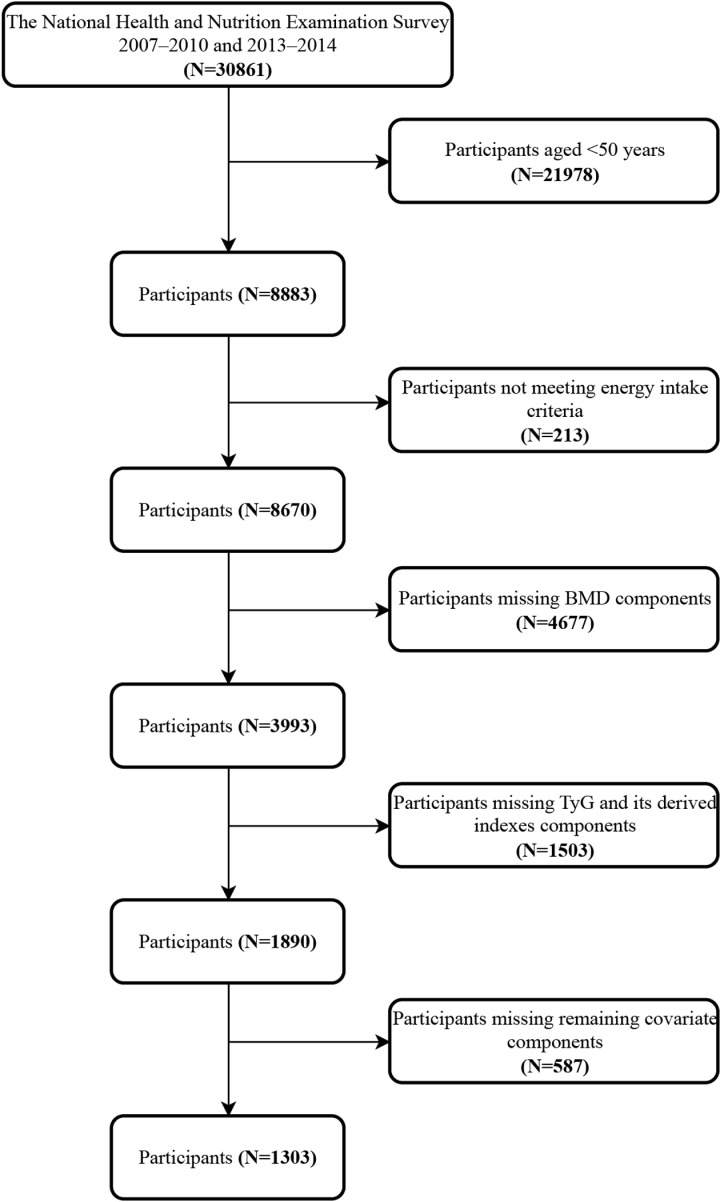
Flowchart for participants’ selection.

All participant information was sourced from the National Health and Nutrition Examination Survey (NHANES), a survey aimed at evaluating the nutritional and health status of the general U.S. population based on a cross-sectional design, updated biennially. Due to the absence of available data on BMD in the NHANES 2011–2012 and 2015–2016 datasets, we ultimately included 1,303 adult participants from the NHANES 2007–2010 and 2013–2014 datasets. The study process is illustrated in Fig 1. Exclusion criteria included: (1) participants under the age of 50 [4]; (2) males with daily caloric intake below 800 kcal/day or above 4,200 kcal/day, females below 500 kcal/day or above 3,500 kcal/day [7,19]; (3) participant missing the TyG index or its combination with obesity indicators; (4) participant lacking BMD indicators; (5) subjects missing other covariates.

### 2.2 Defnitions of TyG, TyG-BMI, TyG-WC, and TyG-WHtR

Data from the NHANES database, including fasting glucose, triglycerides, body mass index, height, and waist circumference, were used to calculate TyG, TyG-BMI, TyG-WC, and TyG-WHtR indices. Blood samples were collected following a minimum 9-hour overnight fast. Fasting triglycerides were quantified using enzymatic methods with Roche Modular P and Roche Cobas 6000 analyzers. Fasting glucose concentrations were determined via hexokinase-mediated reaction using a Roche/Hitachi Cobas C 501 analyzer. All measurement methods are thoroughly documented in the NHANES guidelines. During the calculation of these four indices, we noted that the values of TyG-WC and TyG-BMI were significantly higher than the values of TyG and TyG-WHtR, indicating a substantial difference in numerical ranges. To facilitate comparisons between these four indices and to make them more suitable for clinical application, we have standardized the scaling of TyG-WC and TyG-BMI to bring the ranges of the four indices closer together.

The following are specific formulas for the four indices:


$$\text{TyG}=\text{ln}\left(\frac{\text{fasting triglycerides (mg/dl) }\times \text{fasting glucose (mg/dl)}}{2}\right)$$ (1)

$$\text{WHtR}=\frac{\text{Waist circumference}}{\text{Height}}$$ (2)

$$\text{TyG-WC}=\frac{\text{TyG }\times \text{Waist circumference}}{100}$$ (3)

$$\text{TyG-WHtR}=\frac{\text{TyG }\times \text{Waist circumference}}{\text{Height}}$$ (4)

$$\text{TyG-BMI}=\frac{\text{TyG }\times \text{BMI}}{100}$$ (5)

### 2.3 Definition of osteopenia and osteoporosis

All participants underwent BMD examinations of the femoral neck, lumbar spine, and total hip (intertrochanter) region using dual-energy X-ray absorptiometry (DXA), which was acquired by a Hologic Discovery Model A densitometer (Hologic, Inc., Bedford, Massachusetts) using software version APEX 3. 2. All DXA examination data were analyzed using Hologic APEX software (version 4. 0). More detailed information can be found on the NHANES website. According to guidelines and previous studies, we use T-scores to define osteoporosis and osteopenia. The reference value for BMD is derived from the data of non-Hispanic white adults aged 20–29 years in the NHANES III database [20,21]. If the BMD value was 2. 5 standard deviations or more below the reference value, it was considered osteoporosis; if the BMD value was within 1 standard deviation of the reference value, it was recognized as normal. Individuals with BMD values between 1 and 2.5 standard deviations were identified as having osteopenia.

### 2.4 Covariates considered

Among the covariates considered in this study: age was considered a continuous variable, sex was divided into two categories: male and female, and race was divided into three classifications: non-Hispanic white, non-Hispanic black, and other races. Data for total calcium, blood creatinine, aspartate aminotransferase, alanine aminotransferase, alkaline phosphatase, total cholesterol, and vitamin D, were derived from data on blood samples provided by the participants in the mobile medical center, and these covariates are treated as continuous variables. The AHEI (Alternative Healthy Eating Index) and total energy intake were extracted from data in the Dietary Survey Module. The AHEI, an evolution of the original Healthy Eating Index, was calculated using the methodology proposed by J.J. Zhan et al. [22]. Both are considered to be continuous variables. Considering the impact of genetic factors, we determined whether participants’ parents had a history of osteoporosis and fractures based on their responses in the personal interview section of the survey questionnaire, and also ascertained if the participants themselves had a history of glucocorticoid use, cancer, or diabetes. These covariates were divided into two groups: yes and no. Milk product consumption, obtained from the dietary survey module, is categorized into four groups: never, rarely, sometimes, and often. Education attainment, obtained from the Education Attainment Questionnaire (QEAA), is categorized into two groups: high school or below and college graduate or above [23]. Income level is classified based on the Poverty Income Ratio (PIR), with a PIR < 1 indicating “poor” and a PIR ≥ 1 indicating “not poor”. Smoke status is divided into two categories: never smoker and smoker. Activity level, based on occupational activity, is categorized into Low and high. Alcohol use is classified into non-drinkers and drinkers [23]. For more detailed information about the measurement of covariates, please visit the NHANES website (https://www.cdc.gov/nchs/nhanes/index.htm).

### 2.5 Statistical analysis

We conducted data analysis using the weights recommended by the official NHANES. Weighted mean [95% CI] was used to describe continuous variables, whereas for categorical variables, weighted percentage [95% CI] was used to describe them. During statistical analysis, differences between groups for continuous variables were assessed using the Wilcoxon rank-sum test to accommodate non-normal distribution complex survey data. Comparisons of categorical variables were conducted using the chi-squared test with Rao & Scott’s second-order correction. Participants’ baseline characteristics were categorized according to the quartiles of these indexes. To assess the linear association between the TyG index and its related indexes with BMD, we used weighted linear regression analysis, represented by the Beta coefficient and its 95% confidence interval to indicate the strength of the association. Additionally, weighted logistic regression analysis was used to assess the association between these indexes and the risk of osteopenia/osteoporosis, with results presented as odds ratios (OR) and their 95% confidence intervals. Furthermore, we divided these indexes by quartiles and evaluated their association with BMD and the risk of osteopenia/osteoporosis through weighted linear and logistic regression analysis. To further explore whether this association showed a consistent trend with increasing index levels, we also conducted weighted linear trend tests. To further reveal the dose-response relationship between the TyG index and its related indexes with BMD and the risk of osteopenia/osteoporosis, restricted cubic spline analyses adjusted for all variables were performed. Tests for non-linearity were conducted using the likelihood ratio test. To assess whether the association between these indexes with BMD as well as osteoporosis risk was influenced by other factors, we also conducted stratified analyses, considering factors such as gender, race, alcohol use, educational level, smoking status, parental history of osteoporosis, parental history of fractures, diabetes, cancer, dairy product consumption, activity level, and income level. Additionally, we conducted sensitivity analyses to further verify the robustness of the results, performing separate analyses for males and females. All statistical analyses were conducted by R software version 4.3.1, and results were considered statistically significant when the p-value was below 0.05.

## 3 Results

### 3.1 Analysis of participant characteristics by quartiles of TyG, TyG-BMI, TyG-WC and TyG-WHtR

As shown in Table 1 and S1–S3 Tables, participants with higher TyG, TyG-BMI, TyG-WC, and TyG-WHtR values were more likely to be male, and the prevalence of diabetes was also higher in this group. Furthermore, these participants typically exhibited higher levels of alanine aminotransferase (ALT) and alkaline phosphatase (ALP), lower scores on the Alternative Healthy Eating Index (AHEI), and lower educational attainment.

**Table 1:** Participant Characteristics by Quartiles of TyG (weighted).

Characteristic	TyG (N = 1,303)				P Value^3^
	[6.97, 8.31], N = 327^1^^,^^2^	[8.31, 8.7], N = 326^1^^,^^2^	[8.7, 9.1], N = 324^1^^,^^2^	[9.1, 11.6], N = 326^1^^,^^2^	
**Age, (years)**	61 [60, 62]	61 [60, 63]	61 [60, 62]	62 [61, 63]	0.7
**BMI, (kg/m^2)**	26.5 [26, 27]	28.2 [27, 29]	29.2 [29, 30]	30.1 [30, 31]	**<0.001**
**Calcium, (mmol/L)**	2.35 [2.3, 2.4]	2.34 [2.3, 2.4]	2.35 [2.3, 2.4]	2.37 [2.3, 2.4]	**0.027**
**Creatinine, (mg/dL)**	0.88 [0.83, 0.92]	0.93 [0.89, 0.96]	0.91 [0.88, 0.93]	0.89 [0.85, 0.93]	**0.034**
**AST, (U/L)**	26 [23, 28]	25 [24, 26]	25 [24, 26]	26 [25, 27]	0.3
**ALT, (U/L)**	23 [20, 26]	25 [24, 26 ]	25 [24, 27 ]	27 [25, 29 ]	**<0.001**
**ALP, (IU/L)**	65 [63, 67]	66 [63, 68]	67 [65, 69]	71 [68, 74]	**0.009**
**Cholesterol, (mmol/L)**	5.03 [4.9, 5.2]	5.16 [5.1, 5.3]	5.25 [5.1, 5.4]	5.55 [5.4, 5.8]	**0.003**
**AHEI**	46 [44, 47]	43 [41, 44 ]	40 [39, 42 ]	40 [38, 42 ]	**<0.001**
**Total energy, (kcal/day)**	1,832 [1,741, 1,924]	1,977 [1,852, 2,101]	1,993 [1,868, 2,117]	1,925 [1,828, 2,022 ]	0.2
**Vitamin D, (nmol/L)**	69 [65, 73]	70 [66, 75]	66 [63, 69]	63 [60, 67]	0.066
**Weight, (kg)**	74 [71, 77]	80 [77, 83]	82 [79, 84]	85 [83, 88]	**<0.001**
**Triglycerides, (mg/dL)**	64 [62, 67]	99 [97, 101]	137 [133, 140]	235 [222, 249]	**<0.001**
**Fasting glucose, (mg/dL)**	97 [95, 98]	103 [101, 104]	109 [106, 112]	130 [123, 138]	**<0.001**
**FN BMD, (gm/cm2)**	0.75 [0.73, 0.77]	0.76 [0.73, 0.78]	0.77 [0.76, 0.79]	0.80 [0.78, 0.82]	**0.008**
**TH BMD, (gm/cm2)**	1.06 [1.0, 1.1]	1.09 [1.1, 1.1]	1.12 [1.1, 1.1]	1.14 [1.1, 1.2]	**0.002**
**LS BMD, (gm/cm2)**	0.97 [0.96, 0.99]	0.99 [0.96, 1.0]	1.00 [0.98, 1.0]	1.04 [1.0, 1.1]	**0.003**
**Sex, %**					0.070
Male	34 [28, 42 ]	46 [38, 55]	49 [41, 56]	46 [38, 56]	
Female	66 [58, 72]	54 [45, 62]	51 [44, 59]	54 [44, 62]	
**Race, %**					**<0.001**
Other/multiracial	8.9 [6.4, 12]	14 [9.9, 19]	15 [11, 20 ]	18 [13, 26 ]	
Non-Hispanic Black	15 [11, 20 ]	9.4 [6.3, 14]	6.5 [4.2, 9.8]	5.6 [3.8, 8.1]	
Non-Hispanic White	76 [71, 81]	77 [69, 83]	78 [72, 83]	76 [69, 82]	
**Income level, %**					0.4
Not poor	91 [86, 94]	94 [91, 96]	93 [90, 95]	91 [88, 94]	
Poor	9.0 [5.8, 14]	6.3 [4.3, 8.9]	7.0 [4.9, 9.9]	8.7 [6.0, 12]	
**Alcohol use, %**					0.6
Non drinker	28 [22, 35 ]	29 [22, 38 ]	24 [18, 30 ]	28 [21, 35 ]	
Drinker	72 [65, 78]	71 [62, 78]	76 [70, 82]	72 [65, 79]	
**Education attainment, %**					**<0.001**
High school or below	26 [20, 33 ]	37 [32, 42 ]	40 [32, 48]	51 [43, 58]	
College graduate or above	74 [67, 80]	63 [58, 68]	60 [52, 68]	49 [42, 57]	
**Smoke status, %**					0.070
Never smoker	59 [50, 67]	57 [50, 63]	48 [41, 55]	49 [42, 55]	
Smoker	41 [33, 50]	43 [37, 50]	52 [45, 59]	51 [45, 58]	
**Milk product consumption, %**					**0.048**
Never	24 [18, 31 ]	16 [11, 22 ]	18 [13, 24 ]	12 [8.9, 16]	
Rarely	17 [12, 23 ]	11 [7.9, 16]	12 [7.9, 17]	11 [6.4, 17]	
Sometimes	22 [18, 27 ]	28 [22, 35 ]	27 [22, 34 ]	33 [26, 41 ]	
Often	37 [30, 46]	45 [37, 53]	43 [35, 51]	44 [35, 53]	
**Activity level, %**					0.12
Low	62 [52, 70]	61 [52, 69]	57 [47, 66]	49 [42, 56]	
High	38 [30, 48]	39 [31, 48]	43 [34, 53]	51 [44, 58]	
**Glucocorticoid use, %**	4.7 [2.7, 7.9]	5.8 [3.7, 8.9]	5.2 [2.9, 9.1]	6.2 [4.0, 9.7]	0.9
**Parents with osteoporosis, %**	18 [13, 24 ]	20 [14, 27 ]	22 [15, 31 ]	19 [13, 26 ]	0.8
**Parents with fracture history, %**	11 [7.3, 16]	10 [7.4, 15]	18 [13, 24 ]	13 [8.7, 18]	0.061
**Diabetes, %**	6.1 [3.5, 11]	13 [9.3, 18]	13 [9.0, 18]	32 [26, 38 ]	**<0.001**
**Cancer, %**	18 [13, 24 ]	16 [11, 23 ]	14 [9.8, 20]	16 [12, 21 ]	0.7

^1^Mean; %.

^2^CI = Confidence Interval.

^3^Wilcoxon rank-sum test for complex survey samples; chi-squared test with Rao & Scott’s second-order correction.

### 3.2 The association of TyG, TyG-BMI, TyG-WC, and TyG-WHtR with BMD and risk of osteopenia/osteoporosis

In our study, through weighted multivariate linear regression and multivariate logistic regression analyses, we assessed the correlation between TyG, TyG-BMI, TyG-WC, and TyG-WHtR with BMD as well as the risk of osteopenia/osteoporosis. The results of these analyses are displayed in Tables 2 and 3 respectively. Data in Table 2 show that in the analyses for the femoral neck, lumbar spine, and total hip region, there exists a significant positive correlation between these four indexes and BMD (p < 0.01), This association remains stable in the basic model without adjusted covariates, in Model 2 adjusting for age, sex, and race, and in Model 3 with full adjustment for confounding factors. Furthermore, the multivariate logistic regression analysis results in Table 3 reveal a significant negative correlation between these indexes and the risk of osteopenia/osteoporosis, both in the basic model and in Models 2 and 3 considering other potential influencing factors. This suggests that higher TyG and its related indexes may be associated with a lower risk of osteoporosis/osteopenia. It is noteworthy that among the four indexes, TyG-BMI has the highest association with both BMD and the risk of osteopenia/osteoporosis, especially in the total hip region (Beta 0.137, 95% CI 0.118, 0.156)(OR 0. 150, 95% CI 0. 093, 0. 242).

**Table 2:** Linear regression analysis investigates the association between TyG and its related indexes with BMD (weighted).

Model^1^	Characteristic	Femoral neck BMD			Lumbar spine BMD			Total hip BMD		
		Beta	95% CI^2^	p-value	Beta	95% CI^2^	p-value	Beta	95% CI^2^	p-value
Model1	TyG	0.028	0.010, 0.047	0.004	0.039	0.021, 0.057	<0.001	0.052	0.028, 0.075	<0.001
	TyG.WC	0.032	0.025, 0.039	<0.001	0.034	0.027, 0.041	<0.001	0.050	0.040, 0.060	<0.001
	TyG.BMI	0.097	0.078, 0.116	<0.001	0.090	0.069, 0.111	<0.001	0.137	0.110, 0.164	<0.001
	TyG.WHtR	0.034	0.022, 0.047	<0.001	0.033	0.019, 0.047	<0.001	0.055	0.037, 0.073	<0.001
Model2	TyG	0.029	0.011, 0.046	<0.001	0.038	0.020, 0.056	<0.001	0.046	0.026, 0.065	<0.001
	TyG.WC	0.027	0.021, 0.033	<0.001	0.027	0.019, 0.034	<0.001	0.040	0.031, 0.048	<0.001
	TyG.BMI	0.086	0.070, 0.102	<0.001	0.084	0.066, 0.103	<0.001	0.124	0.104, 0.145	<0.001
	TyG.WHtR	0.038	0.027, 0.048	<0.001	0.036	0.024, 0.048	<0.001	0.059	0.045, 0.073	<0.001
Model3	TyG	0.032	0.012, 0.051	<0.001	0.037	0.020, 0.055	<0.001	0.049	0.029, 0.069	<0.001
	TyG.WC	0.031	0.025, 0.037	<0.001	0.027	0.020, 0.035	<0.001	0.045	0.037, 0.052	<0.001
	TyG.BMI	0.097	0.081, 0.113	<0.001	0.087	0.068, 0.106	<0.001	0.137	0.118, 0.156	<0.001
	TyG.WHtR	0.043	0.033, 0.054	<0.001	0.037	0.025, 0.048	<0.001	0.067	0.054, 0.081	<0.001

^1^Models:

Model 1: Not adjusted.

Model 2: Adjusted Age, Sex, Race.

Model 3: Adjusted Age, Sex, Race, Alcohol use, Education attainment, Smoke status, Glucocorticoid use, Parents with osteoporosis, Parents with fracture history, Creatinine, Calcium, Diabetes, Cancer, AST, ALT, ALP, Cholesterol, Milk product consumption, Activity level, AHEI, Total energy, Vitamin D, Income level.

^2^CI = Confidence Interval.

**Table 3:** Logistic regression analysis investigates the association between TyG and its related indexes with osteopenia/osteoporosis (weighted).

Model^1^	Characteristic	Femoral neck osteopenia/osteoporosis			Lumbar spine osteopenia/osteoporosis			Total hip osteopenia/osteoporosis		
		OR^2^	95% CI^2^	p-value	OR^2^	95% CI^2^	p-value	OR^2^	95% CI^2^	p-value
Model1	TyG	0.653	0.488, 0.875	0.005	0.686	0.519, 0.907	0.009	0.616	0.459, 0.827	0.002
	TyG.WC	0.652	0.581, 0.731	<0.001	0.681	0.609, 0.763	<0.001	0.594	0.507, 0.696	<0.001
	TyG.BMI	0.268	0.195, 0.368	<0.001	0.377	0.282, 0.504	<0.001	0.188	0.124, 0.286	<0.001
	TyG.WHtR	0.644	0.535, 0.775	<0.001	0.715	0.595, 0.860	<0.001	0.493	0.384, 0.632	<0.001
Model2	TyG	0.594	0.415, 0.850	0.003	0.660	0.475, 0.915	0.010	0.574	0.425, 0.776	<0.001
	TyG.WC	0.664	0.591, 0.747	<0.001	0.733	0.654, 0.821	<0.001	0.588	0.501, 0.690	<0.001
	TyG.BMI	0.256	0.183, 0.358	<0.001	0.387	0.290, 0.514	<0.001	0.198	0.133, 0.296	<0.001
	TyG.WHtR	0.557	0.454, 0.683	<0.001	0.663	0.554, 0.792	<0.001	0.449	0.351, 0.576	<0.001
Model3	TyG	0.591	0.395, 0.883	0.006	0.702	0.494, 0.997	0.036	0.573	0.390, 0.841	0.003
	TyG.WC	0.625	0.547, 0.715	<0.001	0.750	0.656, 0.858	<0.001	0.530	0.432, 0.652	<0.001
	TyG.BMI	0.207	0.144, 0.296	<0.001	0.411	0.295, 0.572	<0.001	0.150	0.093, 0.242	<0.001
	TyG.WHtR	0.513	0.406, 0.647	<0.001	0.696	0.563, 0.861	<0.001	0.380	0.276, 0.522	<0.001

^1^Models:

Model 1: Not adjusted.

Model 2: Adjusted Age, Sex, Race.

Model 3: Adjusted Age, Sex, Race, Alcohol use, Education attainment, Smoke status, Glucocorticoid use, Parents with osteoporosis, Parents with fracture history, Creatinine, Calcium, Diabetes, Cancer, AST, ALT, ALP, Cholesterol, Milk product consumption, Activity level, AHEI, Total energy, Vitamin D, Income level.

^2^OR = Odds Ratio, CI = Confidence Interval.

### 3.3 Linear trend analysis of TyG, TyG-BMI, TyG-WC, and TyG-WHtR with BMD and risk of osteopenia/osteoporosis

**Fig 2:**
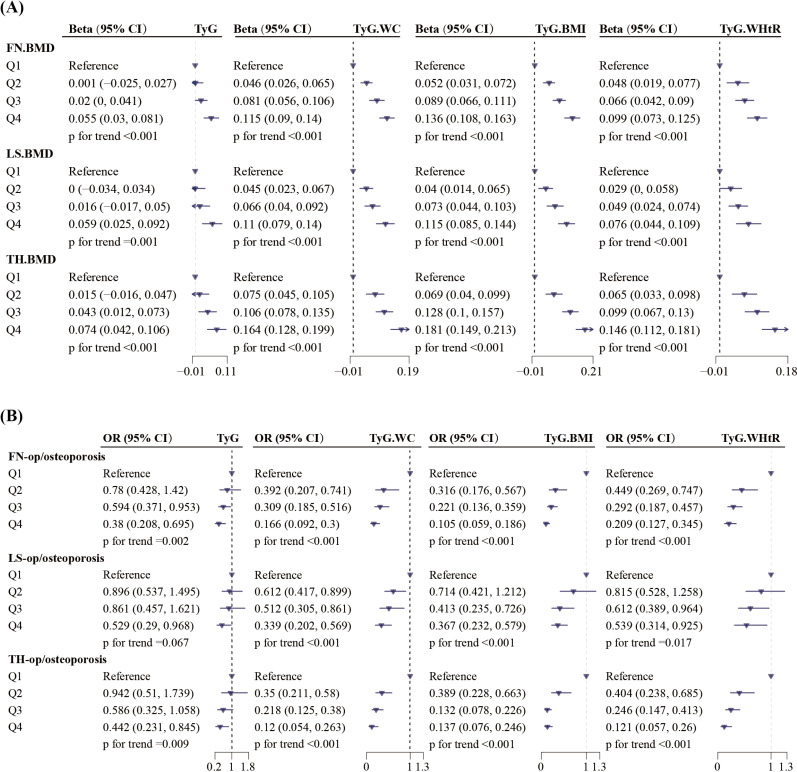
The association between TyG and its related indexes with BMD and osteopenia/osteoporosis (weighted). Adjusted Covariables: Age, Sex, Race, Alcohol use, Education attainment, Smoke status, Glucocorticoid use, Parents with osteoporosis, Parents with fracture history,Creatinine, Calcium, Diabetes, Cancer, AST, ALT, ALP, Cholesterol, Milk product consumption, Activity level, AHEI, Total energy, Vitamin D, Income level.

In our study, we divided TyG, TyG-BMI, TyG-WC, and TyG-WHtR into quartiles (Q1–Q4), transforming these indexes into categorical variables. After adjusting all relevant covariates in weighted linear and logistic regression models, we further explored the association between these indexes and BMD as well as the risk of osteopenia/osteoporosis, with corresponding results presented in Fig 2. As shown in Fig 2A, TyG and its related indexes maintained a significant positive relationship with BMD. Using the first quartile (Q1) as a reference, the positive correlation between them and BMD gradually strengthened with increasing quartiles, exhibiting a statistically significant trend (p for trend < 0.01). Among these indexes, TyG-BMI shows the strongest correlation with BMD in these three regions, followed by TyG-WC. In the highest quartile (Q4), the correlation between TyG-BMI and BMD in the total hip region is most pronounced (Beta 0.181, 95% CI 0.149–0.213). In the results presented in Fig 2B, we observed a significant negative correlation between these indexes and the risk of osteopenia/osteoporosis. Similarly, using the first quartile (Q1) as the baseline, the risk of osteopenia/osteoporosis gradually decreased with rising quartiles, indicating a statistically significant trend. In the highest quartile (Q4), TyG-BMI demonstrates the strongest association with osteopenia/osteoporosis in regions of the femoral neck (OR 0.105, 95% CI 0.059–0.186), followed by TyG-WC. In contrast, TyG-WC has the strongest association with osteopenia/osteoporosis in regions of lumbar spine (OR 0.339, 95% CI 0.202–0.569) and total hip (OR 0.12, 95% CI 0.054–0.263), followed by TyG-BMI.

### 3.4 Restricted cubic spline (RCS) curve analysis of the associations between TyG, TyG-BMI, TyG-WC, and TyG-WHtR with BMD and osteopenia/osteoporosis

**Fig 3:**
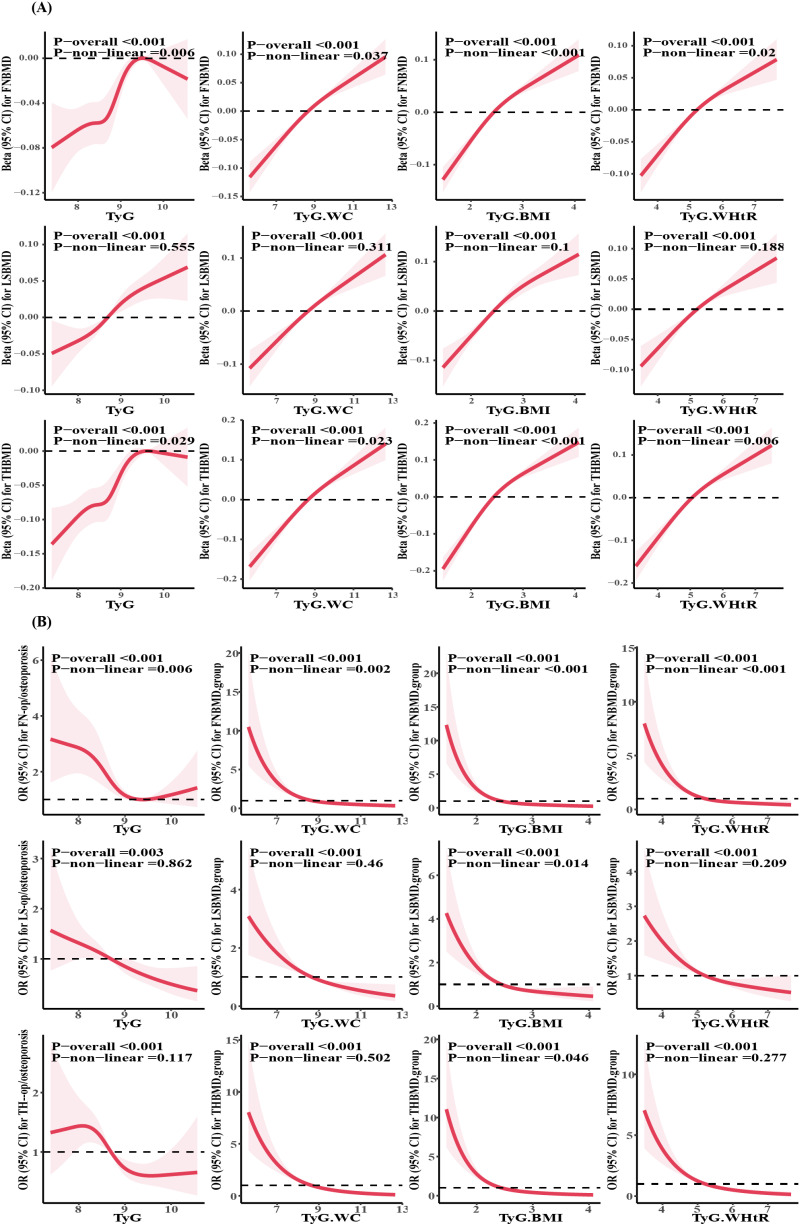
RCS curve of the associations between TyG, TyG-BMI, TyG-WC, and TyG-WHtR with BMD and osteopenia/osteoporosis. Adjusted Covariables: Age, Sex, Race, Alcohol use, Education attainment, Smoke status, Glucocorticoid use, Parents with osteoporosis, Parents with fracture history,Creatinine, Calcium, Diabetes, Cancer, AST, ALT, ALP, Cholesterol, Milk product consumption, Activity level, AHEI, Total energy, Vitamin D, Income level.

To effectively simulate and illustrate the relationships between the TyG, TyG-BMI, TyG-WC, and TyG-WHtR indexes with BMD and the risk of osteopenia/osteoporosis, we utilized restricted cubic spline curves, adjusting for all relevant covariates. As shown in Fig 3A, all four indexes demonstrate a significant linear relationship with lumbar spine BMD (P-overall < 0.001). However, their association with femoral neck and total hip region BMD shows a significant non-linear association (p-non-linear < 0.05), with the increase in BMD slowing down upon reaching certain thresholds for the four indexes. Notably, TyG displayed an “n” shaped curve to the femoral neck and total hip BMD. As shown in Fig 3B, there is a significant non-linear association between the four indexes and the risk of osteopenia/osteoporosis in the femoral neck, characterized by an “L” shaped curve (p-non-linear < 0.05). TyG, TyG-WC, and TyG-WHtR show a linear relationship with the risk of osteopenia/osteoporosis in the lumbar spine and total hip regions (P-overall < 0.05), while TyG-BMI exhibits an “L” shaped non-linear relationship with the risk of osteopenia/osteoporosis in these two areas (p-non-linear < 0.05).

### 3.5 Stratification analysis of TyG, TyG-BMI, TyG-WC, and TyG-WHtR to BMD and osteopenia/osteoporosis (weighted)

After adjusting for all confounding factors, we conducted weighted stratified analyses and interaction effect tests on the associations between TyG, TyG-BMI, TyG-WC, and TyG-WHtR with BMD and risk of osteopenia/osteoporosis, to evaluate which factors affect the correlation between these indexes with BMD and the risk of osteopenia/osteoporosis. The detailed results are shown in S1–S6 Figs. The analysis indicates that the association strength between TyG with BMD and osteopenia/osteoporosis may be influenced by factors such as history of cancer, parental history of osteoporosis, and milk product consumption. Additionally, the association strength between TyG-BMI with BMD and osteopenia/osteoporosis may be influenced by a history of glucocorticoid use, income level, race, parental history of osteoporosis, and milk product consumption. The association strength between TyG-WC with BMD and osteopenia/osteoporosis may be influenced by milk product consumption, race, glucocorticoid use, smoke status, and income level. The association strength between TyG-WHtR with BMD and osteopenia/osteoporosis may be influenced by milk product consumption, glucocorticoid use, and income level.

### 3.6 Sensitivity analysis of the association between TyG, TyG-BMI, TyG-WC, and TyG-WHtR with BMD and osteopenia/osteoporosis

To ensure the reliability of the results, we conducted a sensitivity analysis, separately analyzing male and female participants. Detailed results can be found in S4–S7 Tables. The sensitivity analysis showed that TyG-BMI, TyG-WC, and TyG-WHtR had a positive correlation with BMD in the three regions and a negative correlation with the risk of osteopenia/osteoporosis (p < 0.05), consistent with previous results. Additionally, TyG was significantly positively correlated with BMD at the three sites (p < 0.05) and negatively correlated with the risk of osteopenia/osteoporosis at the total hip (p < 0.05). However, after adjusting for all covariates, there was no significant statistical difference in the risk of osteopenia/osteoporosis at the lumbar spine and femoral neck.

## 4 Discussion

The research analyzed data from the 2007–2010 and 2013–2014 cycles of the NHANES for individuals aged 50 and above, exploring the relationship between TyG, TyG-BMI, TyG-WC, and TyG-WHtR with BMD and osteoporosis/osteopenia. It was discovered that these four indexes are positively associated with BMD and negatively associated with the risk of osteopenia/osteoporosis in the areas of the femoral neck, lumbar spine, and total hip. Among these indexes, TyG-BMI showed the strongest association with BMD and osteoporosis/osteopenia in the femoral neck and total hip region, followed by TyG-WHtR. Likewise, TyG-BMI had the highest correlation with BMD and osteoporosis/osteopenia in the lumbar spine area, followed by TyG. This is consistent with some previous research results. For instance, Wen et al.‘s investigation revealed that an elevated TyG-BMI index correlates positively with BMD, while it inversely relates to the risk of fractures among non-diabetic, middle-aged, and senior Chinese individuals [24].

However, existing researches reveal complex and inconsistent relationships between IR with BMD and osteoporosis. Wang et al. found that after adjusting for all covariates, IR was positively correlated with the risk of osteoporosis in Chinese females with type 2 diabetes [25]. Yoon et al. revealed sex-specific correlations between the TyG index and BMD in a Korean cohort: positive associations with lumbar spine, femoral neck, and total hip BMD in males, but negative associations with femoral neck and total body BMD in females[9]. However, studies on postmenopausal females have yielded contrasting results, revealing a positive association between IR and BMD. Investigations in Tunisia and China demonstrate that IR positively correlates with BMD in postmenopausal females with diabetes [14,26]. Corroborating these findings, a study on non-diabetic postmenopausal females also showed positive associations between IR and various bone parameters. These parameters encompass volumetric BMD (vBMD) of the radius and tibia, trabecular vBMD, and the thickness of both trabecular and cortical bone [27]. These inconsistent research findings may be related to the characteristics of the study populations and the nonlinear relationship between IR and BMD. In our study, the TyG demonstrated an N-shaped association with BMD in the femoral neck region, a pattern also observed by Xu et al. [28]. Their research revealed that β-CTX (a marker of bone resorption) initially increases with rising TyG levels but decreases after reaching an inflection point, indicating reduced bone resorption activity beyond a certain TyG threshold. Simultaneously, while the relationship between TyG and P1NP (a bone formation marker) shows nonlinearity, it maintains a predominantly negative correlation, suggesting decreased osteogenic activity with increasing TyG levels. This bidirectional regulatory model may explain the seemingly contradictory relationships between TyG and BMD reported in previous studies. Different study populations, with their varying distributions of TyG levels, may represent different segments of this nonlinear curve, resulting in divergent association patterns.

Currently, the specific biological mechanisms underlying the positive correlation between the TyG and its related indexes with BMD, as well as the negative correlation with the risk of osteoporosis, are unclear. Considering that a higher TyG index typically implies stronger insulin resistance and a higher degree of obesity, our interpretation primarily focuses on the possible roles of insulin resistance, hyperinsulinemia, and obesity. The following are several possible mechanisms we have considered: Insulin resistance leads to elevated insulin levels through a negative feedback mechanism, thus resulting in hyperinsulinemia [14]. Elevated insulin levels, by activating the Mitogen-Activated Protein Kinase (MAPK) and Phosphoinositide 3-Kinase (PI3K) pathways [29], promote the growth, proliferation, and survival of osteoblasts, leading to an increase in bone mass. Additionally, obesity plays a crucial role in this process. As mechanosensitive cells, osteocytes respond to the increased mechanical load caused by obesity by activating the BMP-Smad pathway and enhancing IGF-I expression [30,31], thus promoting bone formation and remodeling, resulting in denser bones. Furthermore, obesity is often accompanied by an increase in adipose tissue, which in turn increases BMD through various pathways. Firstly, aromatase within adipocytes catalyzes the production of estrogen [32], which directly acts on the estrogen receptors of osteoblasts and osteoclasts [33]. On the other hand, it also increases the sensitivity of bone cells to mechanical stress [34], further increasing BMD. Secondly, adipocytes secrete substantial concentrations of leptin, which exerts dual mechanistic effects on bone metabolism. Primarily, leptin directly acts on osteoblasts to stimulate bone formation. Secondarily, it modulates central neuroendocrine pathways by enhancing the growth hormone-insulin-like growth factor-1 (GH-IGF-1) axis while simultaneously suppressing neuropeptide Y, thereby indirectly contributing to increased bone mass [35]. Finally, the results of RCS curves indicate that as the TyG and its related indexes increase, upon reaching a certain threshold, the rate of BMD increment begins to slow down, and the increment of its protective effect against osteoporosis also decelerates. We speculate that this may be related to the hormones in the body that regulate BMD. As the TyG and related indexes increase, the hormones that promote BMD, such as estrogen and insulin, also increase. However, when these hormones reach a certain concentration, a negative feedback mechanism is triggered, leading to a rise in antagonistic hormones such as glucocorticoids and glucagon. These antagonisms weaken their protective effects on the bones. Considering the complexity of endocrine regulation on skeletal health, further in-depth research is necessary to comprehend the specific mechanisms.

The TyG index, as an effective indicator for assessing insulin resistance, has limited and inconsistent clinical evidence regarding its relationship with BMD. However, our study offers a more detailed exploration, providing new insights into the application of the TyG index and its combination with obesity indices in evaluating BMD and the risk of osteoporosis.

To our knowledge, this study is the first to use the TyG index and its combined indices with obesity markers to simultaneously assess BMD and osteoporosis risk. We particularly focused on individuals aged 50 and above, as they constitute the primary demographic for osteoporosis onset. According to guidelines, the occurrence of osteoporosis exponentially increases after the age of 50, making our study more targeted and clinically valuable.

## 5 Strengths and limitations

The study utilized the NHANES database from the United States, securing high-quality anthropometric and laboratory data, which affirmed the reliability of its findings. A significant advantage of this study was the inclusion of multiple critical covariates with appropriate adjustments, including nutrition-related variables such as total energy intake and AHEI, as well as genetic factors like parental history of osteoporosis and fractures. Furthermore, this study not only simultaneously researched BMD and osteoporosis but also conducted an in-depth exploration of multiple skeletal regions, enhancing the reliability and clinical application potential of the results. However, this study has some limitations. First, due to its cross-sectional study design, we are constrained in exploring the causal relationships between insulin resistance, obesity with BMD, and osteoporosis. The study is based on data from the American population, and whether its findings can be broadly applied to other regions remains to be further observed and researched. Secondly, although adjustments were made for some confounding factors, there may still be unmeasured or residual confounding factors affecting our results. Additionally, our study did not account for the distribution of regional fat mass and fat-free mass, which are important factors that could influence both the TyG index and BMD. Consequently, it’s vital to conduct future longitudinal and prospective studies, along with more thorough data gathering - including assessments of regional fat mass and fat-free mass distribution - to confirm the associations between TyG, TyG-BMI, TyG-WC, and TyG-WHtR with BMD and osteoporosis risk.

## 6 Conclusions

Our research revealed that the TyG and its related indexes are positively correlated with BMD and negatively correlated with the risk of osteoporosis. This result simultaneously verifies the non-linear relationship between insulin resistance and BMD and the risk of osteoporosis. This study provides new insights into the association between TyG and its related indexes with BMD and osteoporosis.

## Supporting information

S1 Table Baseline characteristics according to TyG-BMI quartiles. (DOCX)

S2 Table Baseline characteristics according to TyG-WC quartiles. (DOCX)

S3 Table Baseline characteristics according to TyG-WHtR quartiles. (DOCX)

S4 Table The association between TyG, TyG-BMI, TyG-WC, and TyG-WHtR and BMD in Male. (DOCX)

S5 Table The association between TyG, TyG-BMI, TyG-WC, and TyG-WHtR and BMD in Female. (DOCX)

S6 Table The association between TyG, TyG-BMI, TyG-WC, and TyG-WHtR and osteopenia/osteoporosis in Male. (DOCX)

S7 Table The association between TyG, TyG-BMI, TyG-WC, and TyG-WHtR and osteopenia/osteoporosis in Female. (DOCX)

S1 Fig Stratified analysis to explore the association between TyG, TyG-BMI, TyG-WC, and TyG-WHtR and Femoral neck BMD. (TIF)

S2 Fig Stratified analysis to explore the association between TyG, TyG-BMI, TyG-WC, and TyG-WHtR and Total hip BMD. (TIF)

S3 Fig Stratified analysis to explore the association between TyG, TyG-BMI, TyG-WC, and TyG-WHtR and Lumbar spine BMD. (TIF)

S4 Fig Stratified analysis to explore the association between TyG, TyG-BMI, TyG-WC, and TyG-WHtR and Femoral neck osteopenia/osteoporosis. (TIF)

S5 Fig Stratified analysis to explore the association between TyG, TyG-BMI, TyG-WC, and TyG-WHtR and Total hip osteopenia/osteoporosis. (TIF)

S6 Fig Stratified analysis to explore the association between TyG, TyG-BMI, TyG-WC, and TyG-WHtR and Lumbar spine osteopenia/osteoporosis. (TIF)

## Abbreviations

NHANES: National Health and Nutrition Examination Survey
ALT: Alanine Aminotransferase
AST: Aspartate Aminotransferase
ALP: Alkaline Phosphatase
AHEI: Alternative Healthy Eating Index
BMD: Bone Mineral Density
LS: Lumbar Spine
FN: Femoral Neck
TH: Total hip
LSBMD: Lumbar Spine Bone Mineral Density
FNBMD: Femoral Neck Bone Mineral Density
THBMD: Total hip Bone Mineral Density
FN-op/osteoporosis: Femoral neck osteopenia/osteoporosis
LS-op/osteoporosis: Lumbar spine osteopenia/osteoporosis
TH-op/osteoporosis: Total hip and femoral neck osteopenia/osteoporosis
BMI: Body Mass Index
DXA: Dual-Energy X-ray Absorptiometry
PIR: Poverty Income Ratio
HDL-C: High-Density Lipoprotein Cholesterol
IR: Insulin Resistance
HOMA-IR: Homeostatic Model Assessment for Insulin Resistance
HECT: Hyperinsulinemic-Euglycemic Clamp Technique
LDL-C: Low-Density Lipoprotein Cholesterol
WC: Waist Circumference
WHtR: Waist-to-Height Ratio
TyG: Triglyceride-glucose Index
TyG-BMI: Triglyceride-glucose-Body Mass Index
TyG-WC: Triglyceride-glucose-Waist Circumference
TyG-WHtR: Triglyceride-glucose-Waist-to-Height Ratio
