# Supplementary material for: The association between different insulin resistance indexes and bone health in the elderly
Source: PLoS One. 2025 Feb 11;20(2):e0318356. doi: 10.1371/journal.pone.0318356 (PMC11813086; doi:10.1371/journal.pone.0318356)
Supplement: S1 Table — (DOCX) [file pone.0318356.s001.docx]

|  | **TyG.BMI** (N= 1303) | | | |  |
| --- | --- | --- | --- | --- | --- |
| **Characteristic** | **[1.17,2.1]**, N = 326^12^ | **(2.1,2.44]**, N = 326^12^ | **(2.44,2.79]**, N = 325^12^ | **(2.79,5.08]**, N = 326^12^ | **P Value**^3^ |
| **Age, (years)** | 62 [61, 63] | 62 [61, 63] | 61 [60, 62] | 60 [58, 61] | **0.045** |
| **BMI, (kg/m^2)** | 22.6 [22, 23] | 26.4 [26, 27] | 29.5 [29, 30] | 35.4 [35, 36] | **<0.001** |
| **Calcium, (mmol/L)** | 2.36 [2.3, 2.4] | 2.36 [2.3, 2.4] | 2.36 [2.3, 2.4] | 2.33 [2.3, 2.3] | **0.011** |
| **Creatinine, (mg/dL)** | 0.87 [0.83, 0.92] | 0.91 [0.89, 0.94] | 0.92 [0.87, 0.97] | 0.89 [0.86, 0.92] | **0.019** |
| **AST, (U/L)** | 26 [24, 27] | 25 [24, 26] | 25 [23, 28] | 26 [25, 27] | 0.5 |
| **ALT, (U/L)** | 22 [21, 23] | 24 [22, 26] | 26 [23, 30] | 28 [26, 30] | **<0.001** |
| **ALP, (IU/L)** | 63 [60, 66] | 64 [61, 67] | 69 [66, 72] | 73 [70, 75] | **<0.001** |
| **Cholesterol, (mmol/L)** | 5.19 [5.1, 5.3] | 5.31 [5.1, 5.5] | 5.43 [5.3, 5.6] | 5.02 [4.9, 5.1] | **<0.001** |
| **AHEI** | 46 [44, 48] | 43 [41, 45] | 41 [39, 42] | 40 [38, 42] | **<0.001** |
| **Total energy, (kcal/day)** | 1,922 [1,832, 2,012] | 1,941 [1,853, 2,030] | 1,886 [1,787, 1,984] | 1,971 [1,875, 2,066] | 0.5 |
| **Vitamin D, (nmol/L)** | 74 [69, 78] | 68 [64, 71] | 69 [65, 72] | 60 [57, 63] | **<0.001** |
| **Weight, (kg)** | 64 [62, 66] | 75 [73, 77] | 83 [82, 85] | 98 [96, 100] | **<0.001** |
| **Triglycerides, (mg/dL)** | 84 [79, 90] | 116 [110, 123] | 149 [137, 161] | 170 [157, 182] | **<0.001** |
| **Fasting glucose, (mg/dL)** | 98 [96, 100] | 105 [102, 108] | 109 [105, 113] | 124 [118, 130] | **<0.001** |
| **FN BMD, (gm/cm2)** | 0.70 [0.68, 0.71] | 0.76 [0.74, 0.78] | 0.79 [0.77, 0.81] | 0.84 [0.81, 0.86] | **<0.001** |
| **TH BMD, (gm/cm2)** | 1.00 [0.98, 1.0] | 1.09 [1.1, 1.1] | 1.13 [1.1, 1.2] | 1.19 [1.1, 1.2] | **<0.001** |
| **LS BMD, (gm/cm2)** | 0.93 [0.91, 0.95] | 0.99 [0.97, 1.0] | 1.02 [0.99, 1.0] | 1.06 [1.0, 1.1] | **<0.001** |
| **Sex, %** |  |  |  |  | **0.038** |
| Male | 36 [29, 44] | 52 [45, 59] | 45 [39, 51] | 42 [34, 50] |  |
| Female | 64 [56, 71] | 48 [41, 55] | 55 [49, 61] | 58 [50, 66] |  |
| **Race, %** |  |  |  |  | 0.5 |
| Other/multiracial | 16 [12, 21] | 14 [10, 19] | 12 [9.0, 17] | 13 [9.1, 19] |  |
| Non-Hispanic Black | 6.9 [4.5, 11] | 9.3 [7.0, 12] | 11 [8.0, 16] | 9.8 [6.3, 15] |  |
| Non-Hispanic White | 77 [71, 82] | 77 [71, 82] | 76 [69, 82] | 77 [69, 83] |  |
| **Income level, %** |  |  |  |  | 0.4 |
| Not poor | 91 [86, 95] | 94 [92, 96] | 91 [86, 94] | 92 [89, 95] |  |
| Poor | 8.7 [5.2, 14] | 5.6 [3.8, 8.1] | 9.0 [5.9, 14] | 7.6 [5.4, 11] |  |
| **Alcohol use, %** |  |  |  |  | 0.3 |
| Non drinker | 22 [18, 27] | 29 [22, 38] | 30 [24, 36] | 28 [21, 36] |  |
| Drinker | 78 [73, 82] | 71 [62, 78] | 70 [64, 76] | 72 [64, 79] |  |
| **Education attainment, %** |  |  |  |  | 0.10 |
| High school or below | 30 [24, 38] | 36 [27, 46] | 41 [35, 48] | 43 [36, 51] |  |
| College graduate or above | 70 [62, 76] | 64 [54, 73] | 59 [52, 65] | 57 [49, 64] |  |
| **Smoke status, %** |  |  |  |  | 0.5 |
| Never smoker | 57 [49, 64] | 54 [45, 62] | 54 [47, 61] | 49 [42, 56] |  |
| Smoker | 43 [36, 51] | 46 [38, 55] | 46 [39, 53] | 51 [44, 58] |  |
| **Milk product consumption, %** |  |  |  |  | **0.044** |
| Never | 22 [16, 29] | 15 [9.9, 21] | 14 [9.5, 21] | 20 [15, 26] |  |
| Rarely | 17 [11, 26] | 14 [9.9, 19] | 11 [6.8, 17] | 8.7 [5.3, 14] |  |
| Sometimes | 17 [13, 23] | 28 [22, 34] | 33 [25, 41] | 32 [26, 38] |  |
| Often | 43 [33, 54] | 43 [37, 50] | 42 [34, 51] | 39 [32, 47] |  |
| **Activity level, %** |  |  |  |  | >0.9 |
| Low | 59 [49, 68] | 58 [49, 67] | 57 [49, 65] | 57 [49, 64] |  |
| High | 41 [32, 51] | 42 [33, 51] | 43 [35, 51] | 43 [36, 51] |  |
| **Glucocorticoid use, %** | 4.4 [2.3, 8.4] | 5.0 [3.3, 7.5] | 5.1 [2.9, 8.8] | 7.3 [4.8, 11] | 0.5 |
| **Parents with osteoporosis, %** | 23 [17, 31] | 15 [9.7, 23] | 22 [17, 29] | 17 [12, 25] | 0.2 |
| **Parents with fracture history, %** | 10 [7.0, 15] | 14 [9.4, 20] | 17 [13, 23] | 10 [7.4, 15] | 0.11 |
| **Diabetes, %** | 6.4 [4.0, 10] | 9.6 [6.7, 14] | 14 [11, 19] | 31 [25, 39] | **<0.001** |
| **Cancer, %** | 24 [18, 31] | 17 [12, 23] | 11 [7.6, 16] | 13 [9.4, 17] | **0.004** |
| ^1^Mean; % | | | | | |
| ^2^CI = Confidence Interval | | | | | |
| ^3^Wilcoxon rank-sum test for complex survey samples; chi-squared test with Rao & Scott's second-order correction | | | | | |
