# Supplementary material for: The association between different insulin resistance indexes and bone health in the elderly
Source: PLoS One. 2025 Feb 11;20(2):e0318356. doi: 10.1371/journal.pone.0318356 (PMC11813086; doi:10.1371/journal.pone.0318356)
Supplement: S2 Table — (DOCX) [file pone.0318356.s002.docx]

|  | **TyG.WC** (N= 1303) | | | |  |
| --- | --- | --- | --- | --- | --- |
| **Characteristic** | **[4.76,7.68]**, N = 326^12^ | **(7.68,8.66]**, N = 326^12^ | **(8.66,9.62]**, N = 325^12^ | **(9.62,14.2]**, N = 326^12^ | **P Value**^3^ |
| **Age, (years)** | 62 [60, 63] | 61 [60, 62] | 61 [60, 62] | 61 [60, 62] | 0.8 |
| **BMI, (kg/m^2)** | 23.3 [23, 24] | 26.8 [26, 27] | 29.6 [29, 30] | 34.1 [33, 35] | **<0.001** |
| **Calcium, (mmol/L)** | 2.35 [2.3, 2.4] | 2.36 [2.3, 2.4] | 2.35 [2.3, 2.4] | 2.35 [2.3, 2.4] | 0.15 |
| **Creatinine, (mg/dL)** | 0.84 [0.80, 0.89] | 0.91 [0.88, 0.94] | 0.93 [0.89, 0.98] | 0.91 [0.89, 0.94] | **<0.001** |
| **AST, (U/L)** | 25 [24, 27] | 24 [23, 26] | 26 [24, 29] | 26 [24, 27] | 0.7 |
| **ALT, (U/L)** | 22 [20, 23] | 23 [22, 24] | 28 [24, 31] | 27 [25, 30] | **<0.001** |
| **ALP, (IU/L)** | 62 [60, 65] | 68 [65, 70] | 65 [62, 67] | 73 [71, 76] | **<0.001** |
| **Cholesterol, (mmol/L)** | 5.25 [5.1, 5.4] | 5.37 [5.1, 5.6] | 5.23 [5.0, 5.4] | 5.10 [5.0, 5.2] | **0.034** |
| **AHEI** | 45 [44, 47] | 44 [42, 45] | 41 [39, 43] | 39 [37, 41] | **<0.001** |
| **Total energy, (kcal/day)** | 1,803 [1,708, 1,898] | 1,971 [1,844, 2,097] | 1,977 [1,868, 2,085] | 1,977 [1,885, 2,069] | 0.082 |
| **Vitamin D, (nmol/L)** | 72 [68, 77] | 70 [66, 74] | 67 [63, 71] | 61 [58, 63] | **<0.001** |
| **Weight, (kg)** | 63 [62, 65] | 75 [74, 76] | 84 [82, 86] | 98 [95, 100] | **<0.001** |
| **Triglycerides, (mg/dL)** | 80 [75, 85] | 116 [109, 124] | 137 [126, 148] | 185 [173, 196] | **<0.001** |
| **Fasting glucose, (mg/dL)** | 96 [95, 98] | 103 [101, 105] | 110 [106, 113] | 127 [120, 134] | **<0.001** |
| **FN BMD, (gm/cm2)** | 0.70 [0.69, 0.71] | 0.76 [0.74, 0.78] | 0.80 [0.77, 0.82] | 0.82 [0.80, 0.85] | **<0.001** |
| **TH BMD, (gm/cm2)** | 0.99 [0.97, 1.0] | 1.09 [1.1, 1.1] | 1.14 [1.1, 1.2] | 1.18 [1.1, 1.2] | **<0.001** |
| **LS BMD, (gm/cm2)** | 0.92 [0.91, 0.94] | 0.99 [0.97, 1.0] | 1.02 [1.0, 1.0] | 1.06 [1.0, 1.1] | **<0.001** |
| **Sex, %** |  |  |  |  | **<0.001** |
| Male | 23 [17, 31] | 44 [35, 53] | 56 [50, 62] | 52 [44, 60] |  |
| Female | 77 [69, 83] | 56 [47, 65] | 44 [38, 50] | 48 [40, 56] |  |
| **Race, %** |  |  |  |  | 0.4 |
| Other/multiracial | 15 [11, 20] | 15 [11, 20] | 14 [9.9, 20] | 12 [7.5, 17] |  |
| Non-Hispanic Black | 9.9 [6.8, 14] | 12 [8.4, 16] | 8.1 [5.5, 12] | 7.8 [5.0, 12] |  |
| Non-Hispanic White | 75 [69, 81] | 73 [66, 79] | 78 [71, 83] | 81 [73, 86] |  |
| **Income level, %** |  |  |  |  | 0.6 |
| Not poor | 92 [86, 95] | 92 [89, 95] | 94 [91, 96] | 91 [88, 94] |  |
| Poor | 8.2 [4.7, 14] | 7.8 [5.4, 11] | 6.2 [4.1, 9.1] | 8.8 [6.4, 12] |  |
| **Alcohol use, %** |  |  |  |  | 0.7 |
| Non drinker | 28 [22, 35] | 31 [23, 40] | 25 [19, 32] | 26 [19, 34] |  |
| Drinker | 72 [65, 78] | 69 [60, 77] | 75 [68, 81] | 74 [66, 81] |  |
| **Education attainment, %** |  |  |  |  | **0.017** |
| High school or below | 31 [25, 37] | 35 [28, 42] | 40 [34, 47] | 45 [37, 53] |  |
| College graduate or above | 69 [63, 75] | 65 [58, 72] | 60 [53, 66] | 55 [47, 63] |  |
| **Smoke status, %** |  |  |  |  | **0.029** |
| Never smoker | 59 [52, 65] | 59 [50, 67] | 52 [44, 60] | 44 [38, 51] |  |
| Smoker | 41 [35, 48] | 41 [33, 50] | 48 [40, 56] | 56 [49, 62] |  |
| **Milk product consumption, %** |  |  |  |  | 0.090 |
| Never | 21 [15, 29] | 15 [11, 20] | 18 [13, 25] | 16 [11, 23] |  |
| Rarely | 18 [12, 26] | 14 [9.4, 19] | 11 [6.9, 17] | 9.1 [5.0, 16] |  |
| Sometimes | 21 [15, 28] | 24 [19, 31] | 34 [27, 41] | 30 [23, 39] |  |
| Often | 40 [32, 50] | 47 [40, 54] | 37 [31, 44] | 44 [38, 51] |  |
| **Activity level, %** |  |  |  |  | 0.12 |
| Low | 62 [53, 71] | 59 [52, 66] | 49 [39, 60] | 59 [51, 67] |  |
| High | 38 [29, 47] | 41 [34, 48] | 51 [40, 61] | 41 [33, 49] |  |
| **Glucocorticoid use, %** | 4.4 [2.4, 7.9] | 5.3 [3.4, 8.1] | 4.8 [2.7, 8.4] | 7.2 [4.8, 11] | 0.5 |
| **Parents with osteoporosis, %** | 23 [17, 31] | 18 [12, 26] | 21 [16, 28] | 16 [11, 23] | 0.3 |
| **Parents with fracture history, %** | 13 [9.3, 19] | 11 [8.0, 16] | 14 [9.4, 19] | 13 [9.2, 18] | 0.9 |
| **Diabetes, %** | 5.4 [3.2, 9.1] | 8.5 [5.8, 12] | 17 [13, 23] | 30 [24, 37] | **<0.001** |
| **Cancer, %** | 20 [15, 26] | 18 [13, 24] | 14 [9.4, 19] | 13 [10, 18] | 0.13 |
| ^1^Mean; % | | | | | |
| ^2^CI = Confidence Interval | | | | | |
| ^3^Wilcoxon rank-sum test for complex survey samples; chi-squared test with Rao & Scott's second-order correction | | | | | |
