# Supplementary material for: The association between different insulin resistance indexes and bone health in the elderly
Source: PLoS One. 2025 Feb 11;20(2):e0318356. doi: 10.1371/journal.pone.0318356 (PMC11813086; doi:10.1371/journal.pone.0318356)
Supplement: S3 Table — (DOCX) [file pone.0318356.s003.docx]

|  | **TyG.WHtR** (N= 1303) | | | |  |
| --- | --- | --- | --- | --- | --- |
| **Characteristic** | **[2.94,4.63]**, N = 326^12^ | **(4.63,5.21]**, N = 326^12^ | **(5.21,5.82]**, N = 325^12^ | **(5.82,8.66]**, N = 326^12^ | **P Value**^3^ |
| **Age, (years)** | 61 [60, 62] | 61 [60, 62] | 62 [61, 64] | 61 [60, 62] | **0.043** |
| **BMI, (kg/m^2)** | 23.3 [23, 24] | 27.0 [27, 27] | 29.9 [29, 30] | 34.7 [34, 35] | **<0.001** |
| **Calcium, (mmol/L)** | 2.35 [2.3, 2.4] | 2.36 [2.3, 2.4] | 2.36 [2.3, 2.4] | 2.34 [2.3, 2.4] | 0.14 |
| **Creatinine, (mg/dL)** | 0.89 [0.84, 0.93] | 0.92 [0.89, 0.94] | 0.91 [0.87, 0.96] | 0.88 [0.85, 0.90] | **0.001** |
| **AST, (U/L)** | 25 [24, 27] | 26 [24, 27] | 25 [23, 28] | 26 [25, 27] | 0.3 |
| **ALT, (U/L)** | 22 [21, 23] | 25 [23, 27] | 26 [23, 29] | 27 [25, 29] | **0.001** |
| **ALP, (IU/L)** | 62 [60, 65] | 65 [63, 67] | 69 [66, 71] | 73 [69, 76] | **<0.001** |
| **Cholesterol, (mmol/L)** | 5.21 [5.1, 5.3] | 5.22 [5.1, 5.4] | 5.38 [5.2, 5.6] | 5.13 [5.0, 5.3] | **0.027** |
| **AHEI** | 46 [44, 48] | 43 [41, 45] | 39 [38, 41] | 40 [38, 42] | **<0.001** |
| **Total energy, (kcal/day)** | 1,976 [1,870, 2,081] | 1,925 [1,842, 2,008] | 1,915 [1,811, 2,018] | 1,899 [1,778, 2,020] | 0.6 |
| **Vitamin D, (nmol/L)** | 72 [68, 76] | 70 [66, 75] | 65 [62, 68] | 60 [58, 63] | **<0.001** |
| **Weight, (kg)** | 67 [65, 68] | 78 [75, 80] | 84 [82, 86] | 94 [92, 97] | **<0.001** |
| **Triglycerides, (mg/dL)** | 81 [76, 86] | 115 [107, 123] | 149 [138, 160] | 183 [170, 196] | **<0.001** |
| **Fasting glucose, (mg/dL)** | 97 [96, 99] | 105 [102, 108] | 109 [107, 112] | 127 [119, 135] | **<0.001** |
| **FN BMD, (gm/cm2)** | 0.72 [0.71, 0.74] | 0.78 [0.76, 0.80] | 0.77 [0.76, 0.79] | 0.81 [0.78, 0.83] | **<0.001** |
| **TH BMD, (gm/cm2)** | 1.03 [1.0, 1.0] | 1.11 [1.1, 1.1] | 1.12 [1.1, 1.1] | 1.16 [1.1, 1.2] | **<0.001** |
| **LS BMD, (gm/cm2)** | 0.96 [0.94, 0.98] | 1.00 [0.98, 1.0] | 1.00 [0.98, 1.0] | 1.03 [1.0, 1.1] | **0.002** |
| **Sex, %** |  |  |  |  | 0.054 |
| Male | 41 [33, 49] | 51 [44, 59] | 46 [39, 54] | 36 [29, 43] |  |
| Female | 59 [51, 67] | 49 [41, 56] | 54 [46, 61] | 64 [57, 71] |  |
| **Race, %** |  |  |  |  | 0.5 |
| Other/multiracial | 12 [8.6, 17] | 13 [8.6, 19] | 17 [11, 25] | 14 [9.7, 20] |  |
| Non-Hispanic Black | 9.6 [6.6, 14] | 12 [8.2, 16] | 7.5 [5.1, 11] | 8.4 [5.3, 13] |  |
| Non-Hispanic White | 78 [73, 83] | 76 [68, 82] | 76 [67, 83] | 78 [70, 84] |  |
| **Income level, %** |  |  |  |  | 0.12 |
| Not poor | 91 [86, 95] | 95 [93, 97] | 92 [89, 95] | 90 [86, 92] |  |
| Poor | 8.5 [5.0, 14] | 4.9 [3.3, 7.1] | 7.5 [5.0, 11] | 10 [7.5, 14] |  |
| **Alcohol use, %** |  |  |  |  | 0.2 |
| Non drinker | 21 [17, 25] | 29 [21, 38] | 30 [23, 37] | 31 [23, 40] |  |
| Drinker | 79 [75, 83] | 71 [62, 79] | 70 [63, 77] | 69 [60, 77] |  |
| **Education attainment, %** |  |  |  |  | **<0.001** |
| High school or below | 27 [21, 34] | 33 [27, 40] | 47 [41, 54] | 47 [39, 54] |  |
| College graduate or above | 73 [66, 79] | 67 [60, 73] | 53 [46, 59] | 53 [46, 61] |  |
| **Smoke status, %** |  |  |  |  | 0.3 |
| Never smoker | 58 [50, 66] | 54 [47, 61] | 49 [42, 56] | 51 [44, 58] |  |
| Smoker | 42 [34, 50] | 46 [39, 53] | 51 [44, 58] | 49 [42, 56] |  |
| **Milk product consumption, %** |  |  |  |  | 0.11 |
| Never | 19 [13, 26] | 18 [13, 24] | 17 [12, 23] | 18 [12, 26] |  |
| Rarely | 19 [13, 28] | 11 [6.9, 17] | 8.6 [5.2, 14] | 11 [7.5, 17] |  |
| Sometimes | 20 [15, 26] | 28 [22, 35] | 32 [26, 39] | 30 [25, 37] |  |
| Often | 42 [34, 51] | 43 [36, 50] | 42 [34, 51] | 40 [33, 48] |  |
| **Activity level, %** |  |  |  |  | >0.9 |
| Low | 56 [46, 66] | 58 [50, 66] | 59 [50, 67] | 57 [50, 65] |  |
| High | 44 [34, 54] | 42 [34, 50] | 41 [33, 50] | 43 [35, 50] |  |
| **Glucocorticoid use, %** | 3.4 [1.7, 6.7] | 7.3 [4.8, 11] | 2.4 [1.3, 4.6] | 8.6 [5.8, 13] | **0.008** |
| **Parents with osteoporosis, %** | 21 [15, 29] | 17 [12, 25] | 25 [19, 31] | 15 [10, 22] | 0.2 |
| **Parents with fracture history, %** | 10 [7.1, 15] | 15 [10, 21] | 15 [10, 20] | 12 [7.9, 18] | 0.5 |
| **Diabetes, %** | 4.7 [2.9, 7.5] | 11 [7.3, 15] | 16 [12, 22] | 32 [26, 40] | **<0.001** |
| **Cancer, %** | 21 [16, 28] | 16 [11, 23] | 13 [8.9, 19] | 13 [9.1, 19] | 0.12 |
| ^1^Mean; % | | | | | |
| ^2^CI = Confidence Interval | | | | | |
| ^3^Wilcoxon rank-sum test for complex survey samples; chi-squared test with Rao & Scott's second-order correction | | | | | |
