# Supplementary material for: The association between different insulin resistance indexes and bone health in the elderly
Source: PLoS One. 2025 Feb 11;20(2):e0318356. doi: 10.1371/journal.pone.0318356 (PMC11813086; doi:10.1371/journal.pone.0318356)
Supplement: S6 Table — (DOCX) [file pone.0318356.s006.docx]

**The association between TyG, TyG-BMI, TyG-WC, and TyG-WHtR and osteopenia/osteoporosis**

**in Male**

|  | | **Femoral neck osteopenia/osteoporosis** | | | **Total hip osteopenia/osteoporosis** | | | **Lumbar spine osteopenia/osteoporosis** | | |
| --- | --- | --- | --- | --- | --- | --- | --- | --- | --- | --- |
| **Model** | **Characteristic**^1^ | **OR**^2^ | **95% CI**^2^ | **p-value** | **OR**^2^ | **95% CI**^2^ | **p-value** | **OR**^2^ | **95% CI**^2^ | **p-value** |
| Model 1 | TyG | 0.645 | 0.412, 1.01 | 0.056 | 0.509 | 0.328, 0.790 | **0.003** | 0.608 | 0.392, 0.944 | **0.027** |
|  | TyG.WC | 0.674 | 0.552, 0.822 | **<0.001** | 0.622 | 0.496, 0.779 | **<0.001** | 0.768 | 0.646, 0.914 | **0.004** |
|  | TyG.BMI | 0.213 | 0.114, 0.397 | **<0.001** | 0.133 | 0.063, 0.281 | **<0.001** | 0.365 | 0.220, 0.605 | **<0.001** |
|  | TyG.WHtR | 0.571 | 0.406, 0.803 | **0.002** | 0.454 | 0.321, 0.643 | **<0.001** | 0.672 | 0.495, 0.913 | **0.012** |
| Model 2 | TyG | 0.636 | 0.391, 1.03 | 0.066 | 0.504 | 0.315, 0.808 | **0.005** | 0.548 | 0.335, 0.898 | **0.018** |
|  | TyG.WC | 0.635 | 0.515, 0.782 | **<0.001** | 0.589 | 0.463, 0.749 | **<0.001** | 0.752 | 0.628, 0.902 | **0.003** |
|  | TyG.BMI | 0.215 | 0.114, 0.409 | **<0.001** | 0.139 | 0.065, 0.297 | **<0.001** | 0.357 | 0.209, 0.609 | **<0.001** |
|  | TyG.WHtR | 0.511 | 0.353, 0.740 | **<0.001** | 0.408 | 0.278, 0.597 | **<0.001** | 0.634 | 0.460, 0.873 | **0.006** |
| Model 3 | TyG | 0.662 | 0.386, 1.14 | 0.13 | 0.555 | 0.316, 0.976 | **0.042** | 0.626 | 0.338, 1.16 | 0.13 |
|  | TyG.WC | 0.561 | 0.435, 0.725 | **<0.001** | 0.543 | 0.397, 0.741 | **<0.001** | 0.765 | 0.597, 0.981 | **0.036** |
|  | TyG.BMI | 0.141 | 0.066, 0.305 | **<0.001** | 0.103 | 0.040, 0.267 | **<0.001** | 0.353 | 0.180, 0.695 | **0.004** |
|  | TyG.WHtR | 0.427 | 0.272, 0.672 | **<0.001** | 0.354 | 0.208, 0.602 | **<0.001** | 0.640 | 0.412, 0.994 | **0.047** |
| ^1^Models: Model 1: Not adjusted Model 2: Adjusted Age, Race Model 3: Adjusted Age, Race, Alcohol use, Education attainment, Smoke status, Glucocorticoid use, Parents with osteoporosis, Parents with fracture history, Creatinine, Calcium, Diabetes, Cancer, AST, ALT, ALP, Cholesterol, Milk product consumption, Activity level, AHEI, Total energy, Vitamin D, Income level | | | | | | | | | | |
| ^2^OR = Odds Ratio, CI = Confidence Interval | | | | | | | | | | |
