# Supplementary figures and images for: The association between different insulin resistance indexes and bone health in the elderly
Source: PLoS One. 2025 Feb 11;20(2):e0318356. doi: 10.1371/journal.pone.0318356 (PMC11813086; doi:10.1371/journal.pone.0318356)

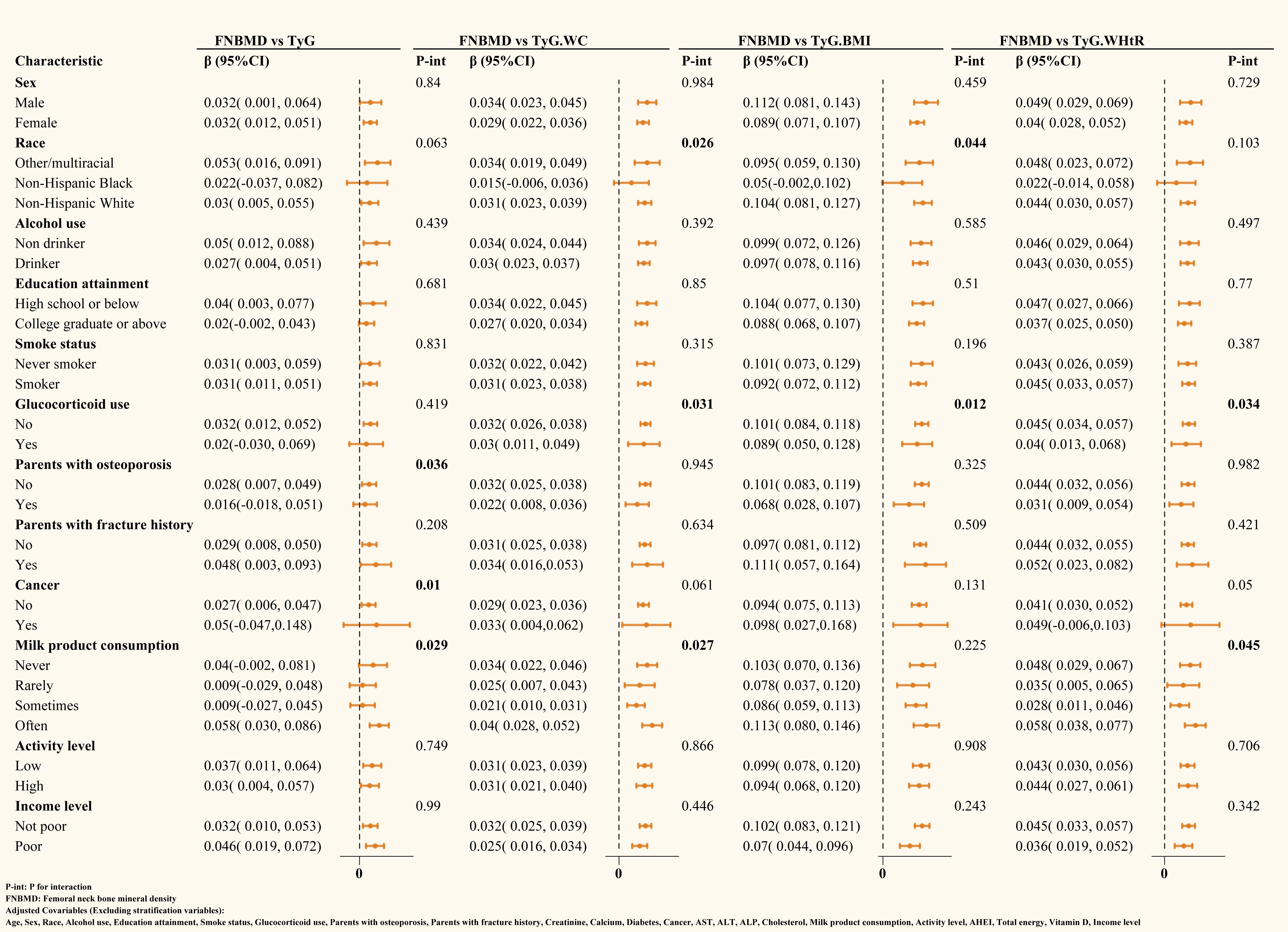

Supplement: S1 Fig — (TIF) [file pone.0318356.s008.tif]

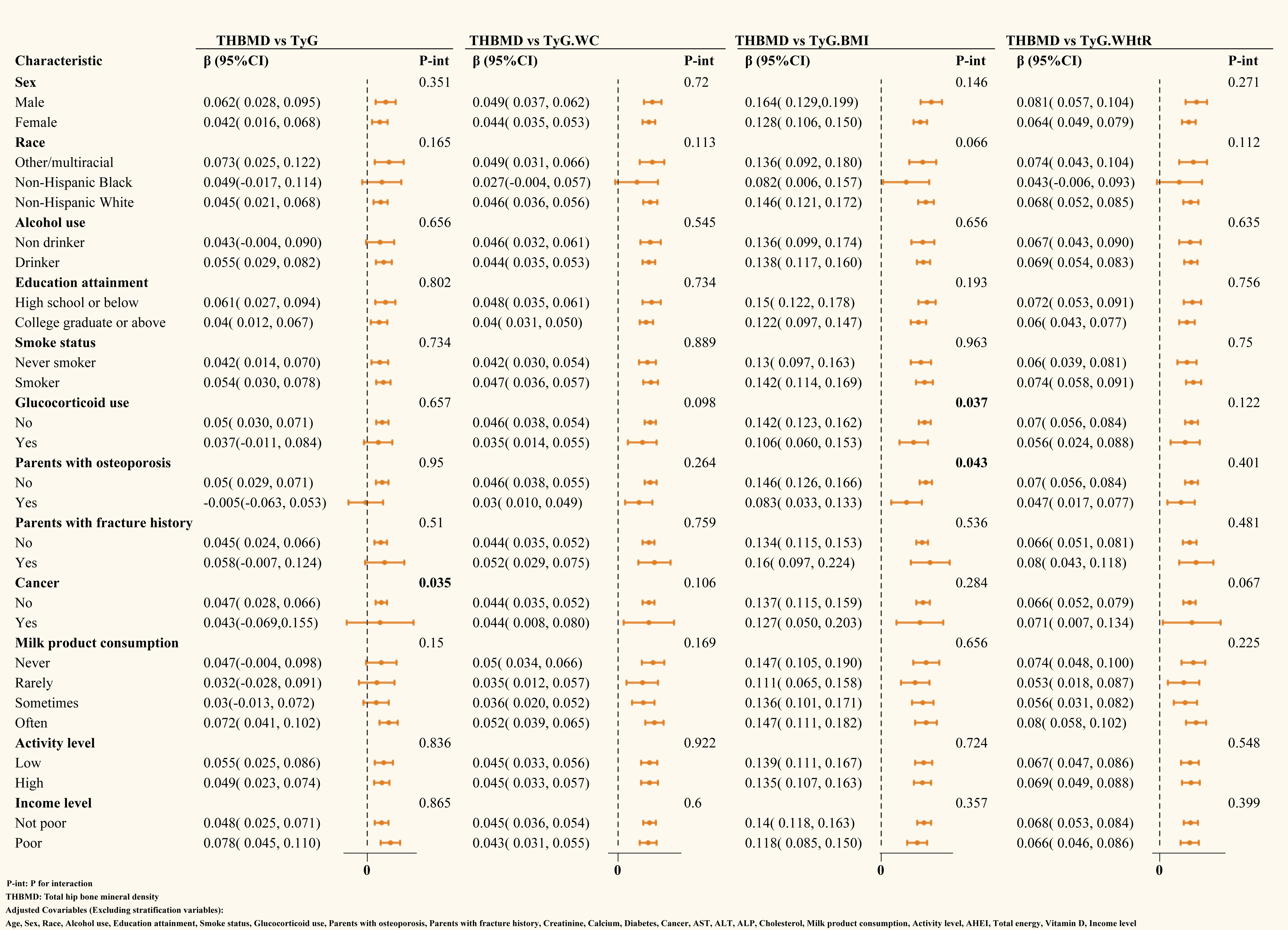

Supplement: S2 Fig — (TIF) [file pone.0318356.s009.tif]

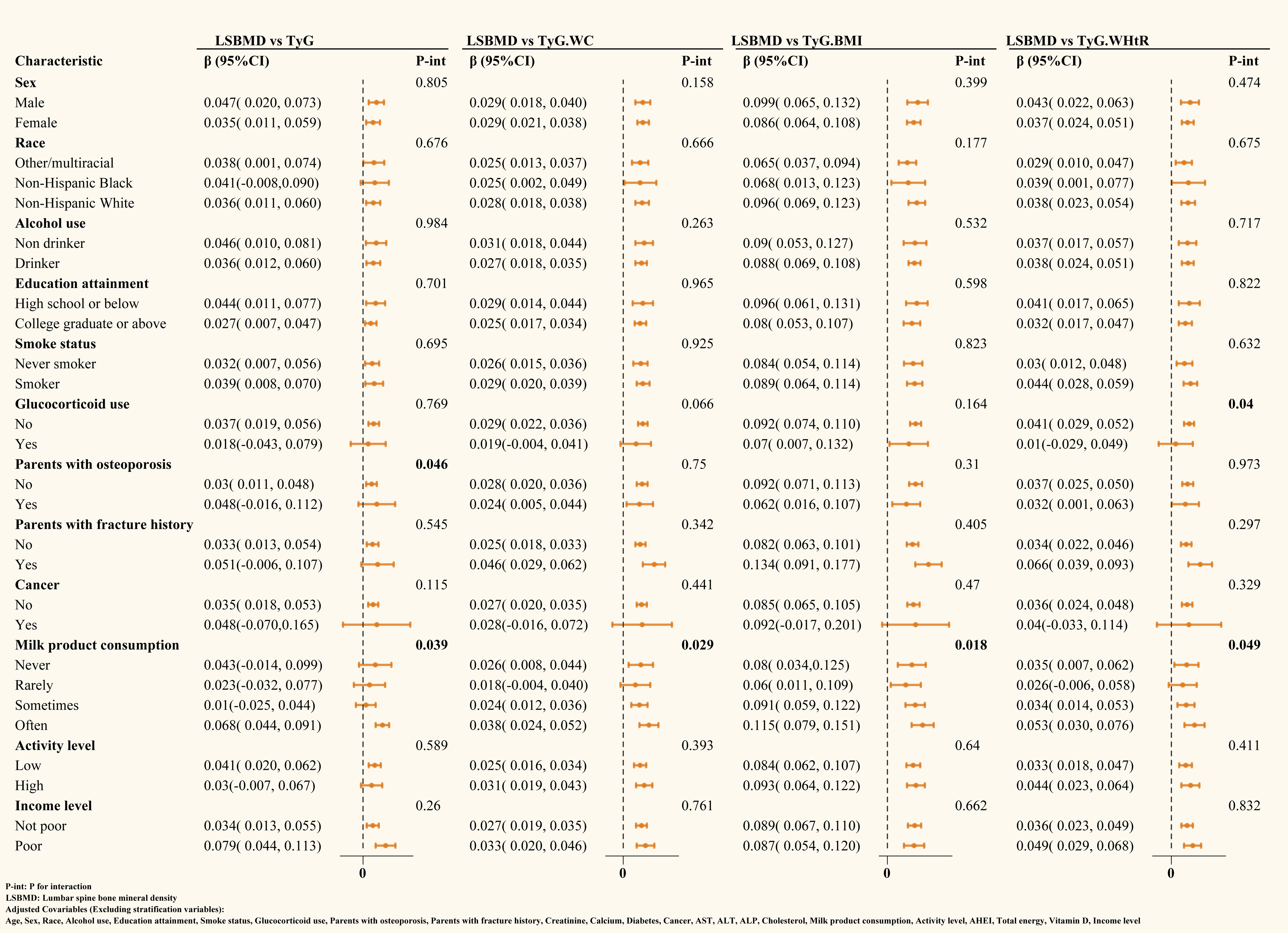

Supplement: S3 Fig — (TIF) [file pone.0318356.s010.tif]

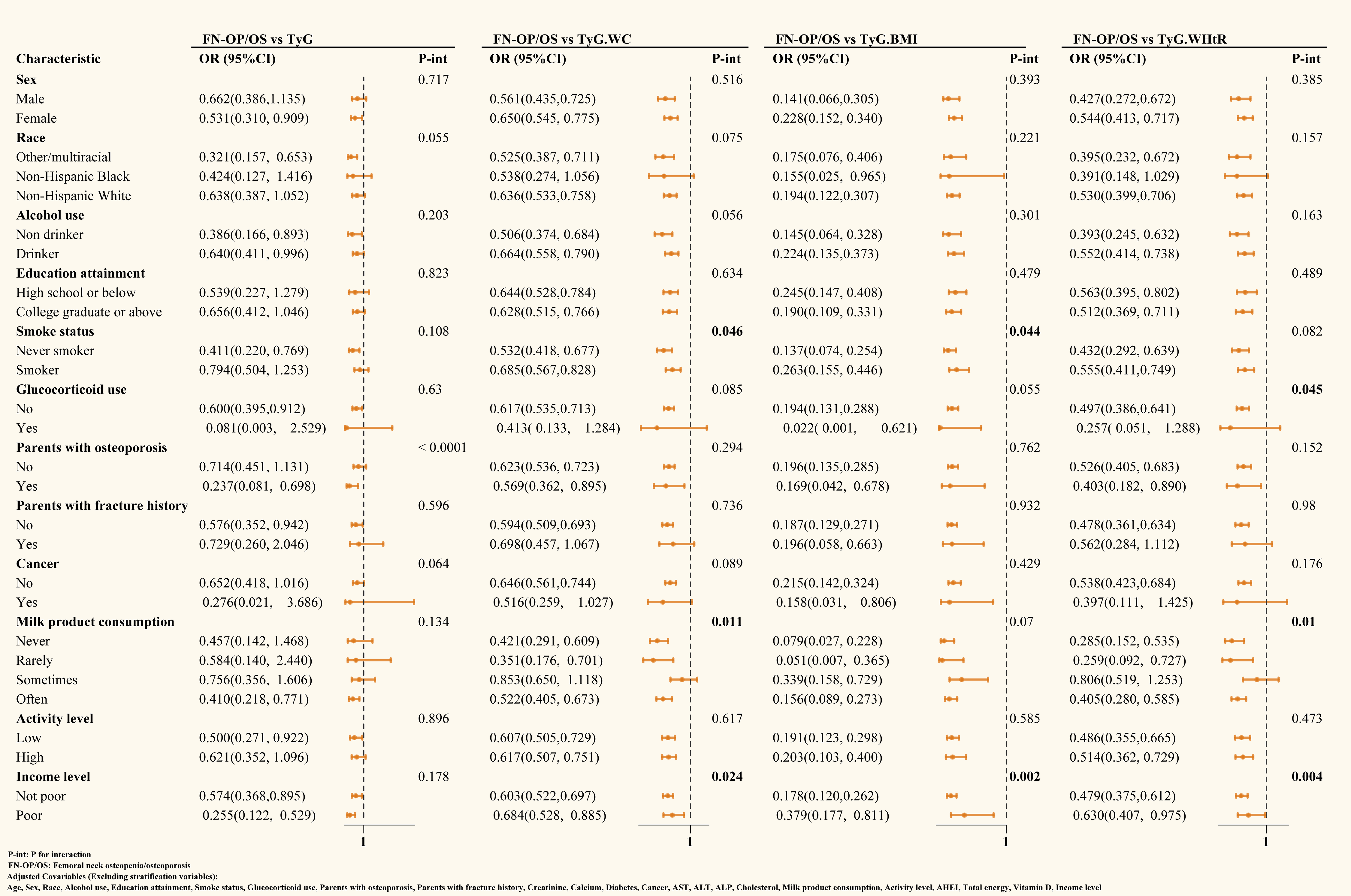

Supplement: S4 Fig — (TIF) [file pone.0318356.s011.tif]

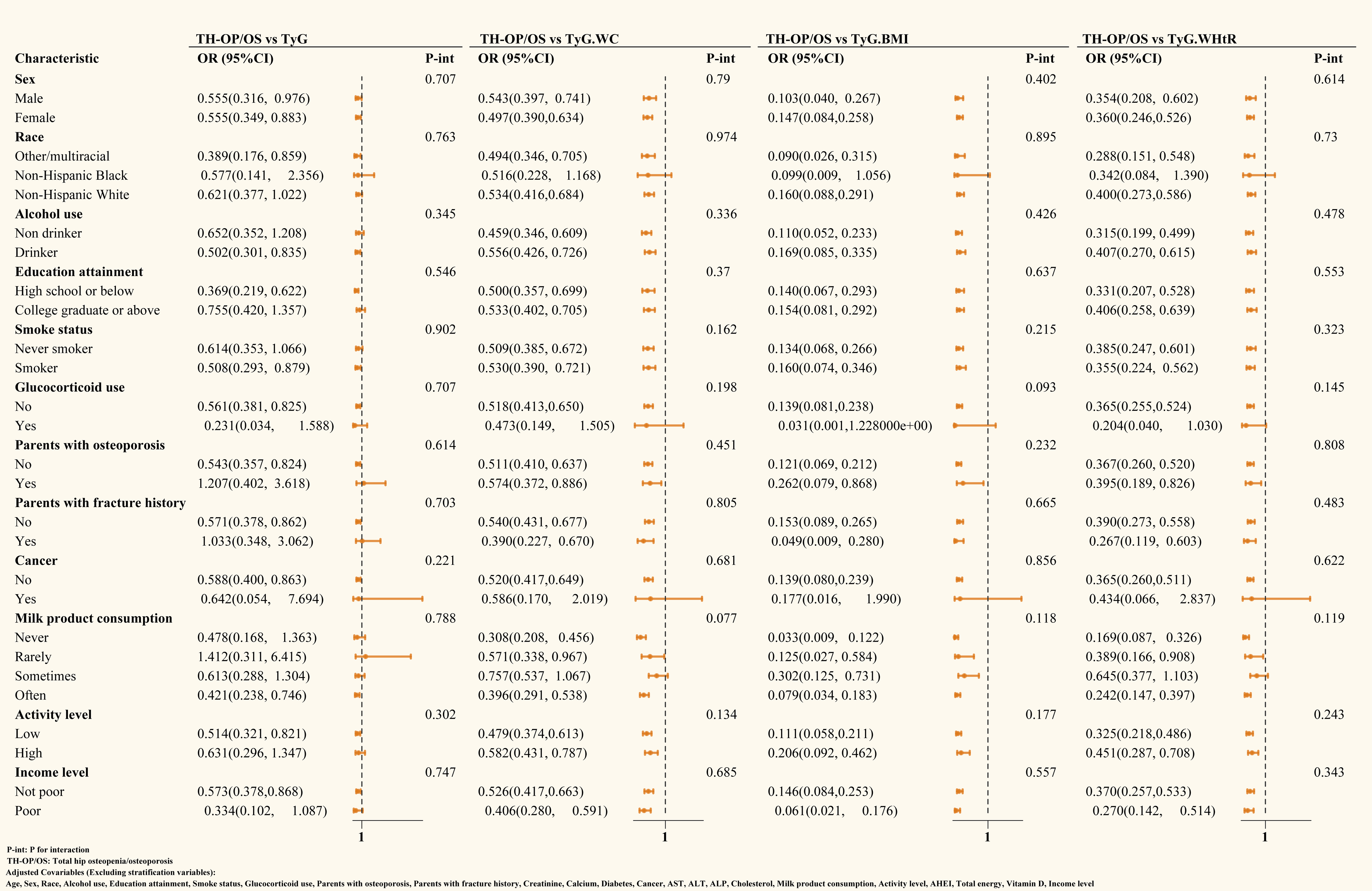

Supplement: S5 Fig — (TIF) [file pone.0318356.s012.tif]

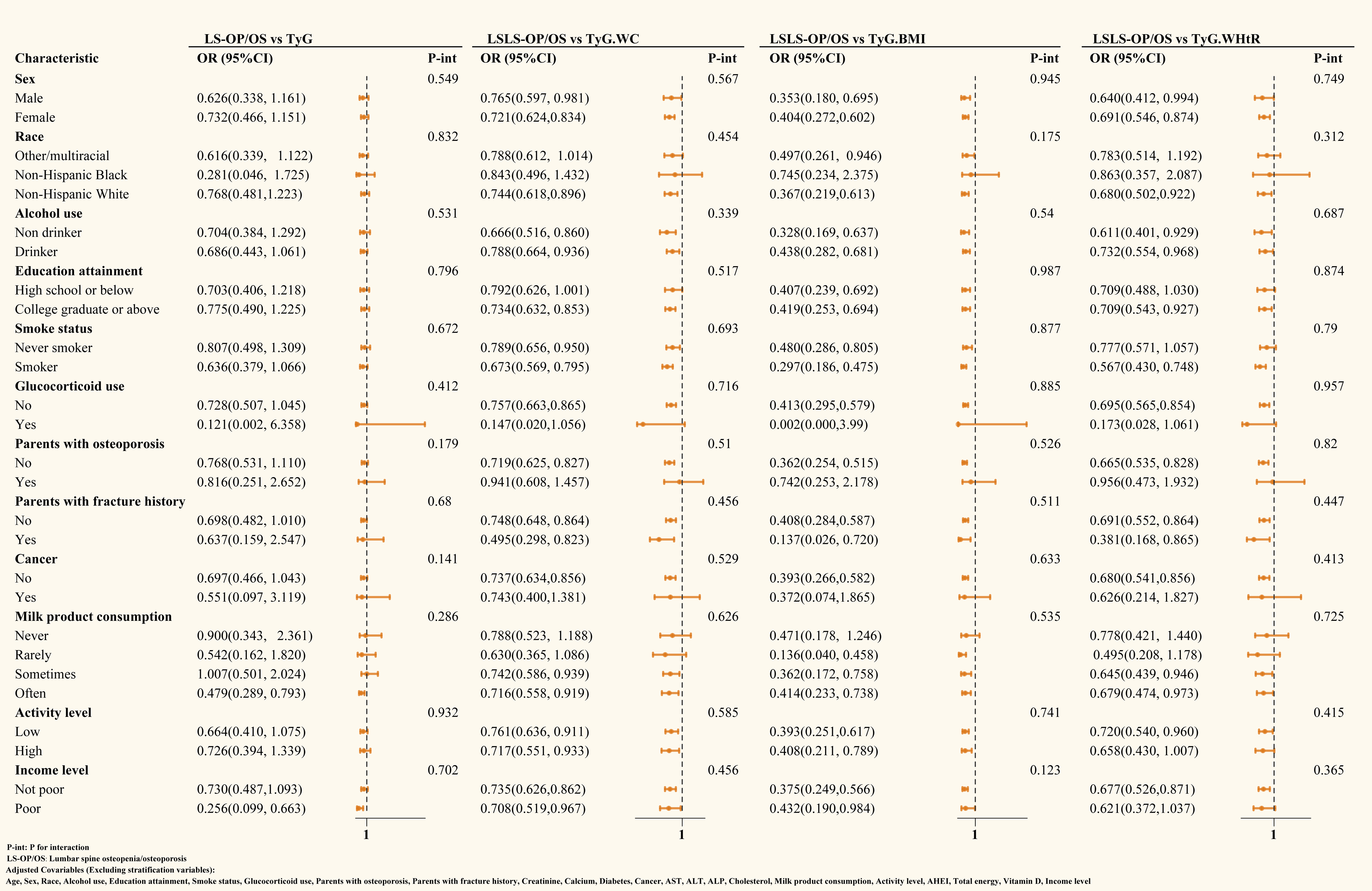

Supplement: S6 Fig — (TIF) [file pone.0318356.s013.tif]
